# Revealing particle-scale powder spreading dynamics in powder-bed-based additive manufacturing process by high-speed x-ray imaging

**DOI:** 10.1038/s41598-018-33376-0

**Published:** 2018-10-10

**Authors:** Luis I. Escano, Niranjan D. Parab, Lianghua Xiong, Qilin Guo, Cang Zhao, Kamel Fezzaa, Wes Everhart, Tao Sun, Lianyi Chen

**Affiliations:** 1 Department of Mechanical and Aerospace Engineering, Missouri University of Science and Technology, Rolla, MO 65409 USA; 2 Department of Materials Science and Engineering, Missouri University of Science and Technology, Rolla, MO 65409 USA; 3 X-ray Science Division, Advanced Photon Source, Argonne National Laboratory, Argonne, IL 60439 USA; 4 Department of Energy’s Kansas City National Security Campus Managed by Honeywell FM&T, Kansas City, MO 64147 USA

## Abstract

Powder spreading is a key step in the powder-bed-based additive manufacturing process, which determines the quality of the powder bed and, consequently, affects the quality of the manufactured part. However, powder spreading behavior under additive manufacturing condition is still not clear, largely because of the lack of particle-scale experimental study. Here, we studied particle-scale powder dynamics during the powder spreading process by using *in-situ* high-speed high-energy x-ray imaging. Evolution of the repose angle, slope surface speed, slope surface roughness, and the dynamics of powder clusters at the powder front were revealed and quantified. Interactions of the individual metal powders, with boundaries (substrate and container wall), were characterized, and coefficients of friction between the powders and boundaries were calculated. The effects of particle size on powder flow dynamics were revealed. The particle-scale powder spreading dynamics, reported here, are important for a thorough understanding of powder spreading behavior in the powder-bed-based additive manufacturing process, and are critical to the development and validation of models that can more accurately predict powder spreading behavior.

## Introduction

In the powder-bed-based additive manufacturing process, powders are spread in a thin layer and then selectively melted/sintered by a heat source or selectively joined together by a liquid binding agent to form a part^1–3^. The quality of the powder bed is known to be one of the main factors that influence the quality of the part being manufactured^4^. For example, in powder bed fusion process, it has been demonstrated that improving the powder bed density and homogeneity decreases melting defects (such as denudation and porosity), and improves the final quality of the part^5–7^. Thus, it is important to study and understand the spreading process in order to accurately predict powder bed quality^8^.

Avalanche testing instruments and rheometers have been used to study powder flowability^9–12^. However, the powder flow environment in avalanche testing instruments and rheometers is very different from the conditions in additive manufacturing. Since powder flow behavior strongly depends on surrounding conditions, it is important to study and understand the flow behavior of powder under additive manufacturing conditions.

Continuous models have been developed to simulate and study the spreading process. Treating the powder as a continuous non-dense material that is subjected to shearing stresses, similar to the ones found during the spreading process, provided an insight into the external forces that affect the powder. With this assumption, the influence of layer thickness, roller geometry, and initial powder properties on the compacted powders’ relative density were studied^13,14^. However, as the powder was not a continuous material (but a collection of interacting individual elements) that interacted with the environment as well, predictions of powder segregation and powder bed surface roughness require study at the particle scale.

The discrete element method (DEM) has become the main tool to computationally study the particle scale spreading process^15^. The influence of the spreading speed, coater type, and powder size distribution on the surface roughness and compaction of the powder bed have been studied by DEM^16–20^. Recent simulation works have used experimentally measured powder size/shape distribution as input for simulation, which improves the accuracy of the DEM model^16,17,19,21^. DEM results showed that defects (voids, surface roughness, thickness non-uniformity) in the powder bed can lead to defects (surface roughness and porosity) in the final manufactured parts^22,23^.

At the current state, the developed DEM models are based on many physical assumptions related to the mechanical properties that govern the dynamic and mechanical behavior of the powder. Experimental characterization of the particle-scale dynamics of powder spreading process under additive manufacturing conditions is critical to validating DEM models and uncovering powder spreading behavior in the powder-bed-based additive manufacturing process. However, this is very challenging for metals due to the lack of transparency of metals to visible light and microscale interaction. Very recently, Chen *et al*. measured the dynamic repose angle during the powder spreading process by using a visible light camera^18^. However, the dynamics of particle-scale powder spreading was not revealed, due to the limited resolution and penetration depth of conventional characterization tools. Very recently, our team, as well as other research groups, have demonstrated that high-energy x-ray imaging could penetrate metals to study powder spattering, melting, and pore evolution during metal additive manufacturing process^24–28^, indicating that x-ray imaging could be a powerful tool for studying powder spreading process.

In this research, we developed an experimental method based on high-speed high-energy x-ray imaging to characterize the powder spreading process with high spatial and temporal resolution and study the particle scale spreading process *in-situ*. With this newly developed experimental approach, we revealed the evolution of the repose angle, the slope surface flow speed, and slope surface roughness during the powder spreading process. We observed and analyzed the evolution and flow of two different types of powder clusters on the slope surface, and closely examined the particles that formed such clusters. We analyzed the dynamic interaction of individual particles with their respective boundaries and calculated coefficients of friction between the powders and boundaries.

## Experimental Approach

**Figure 1:**
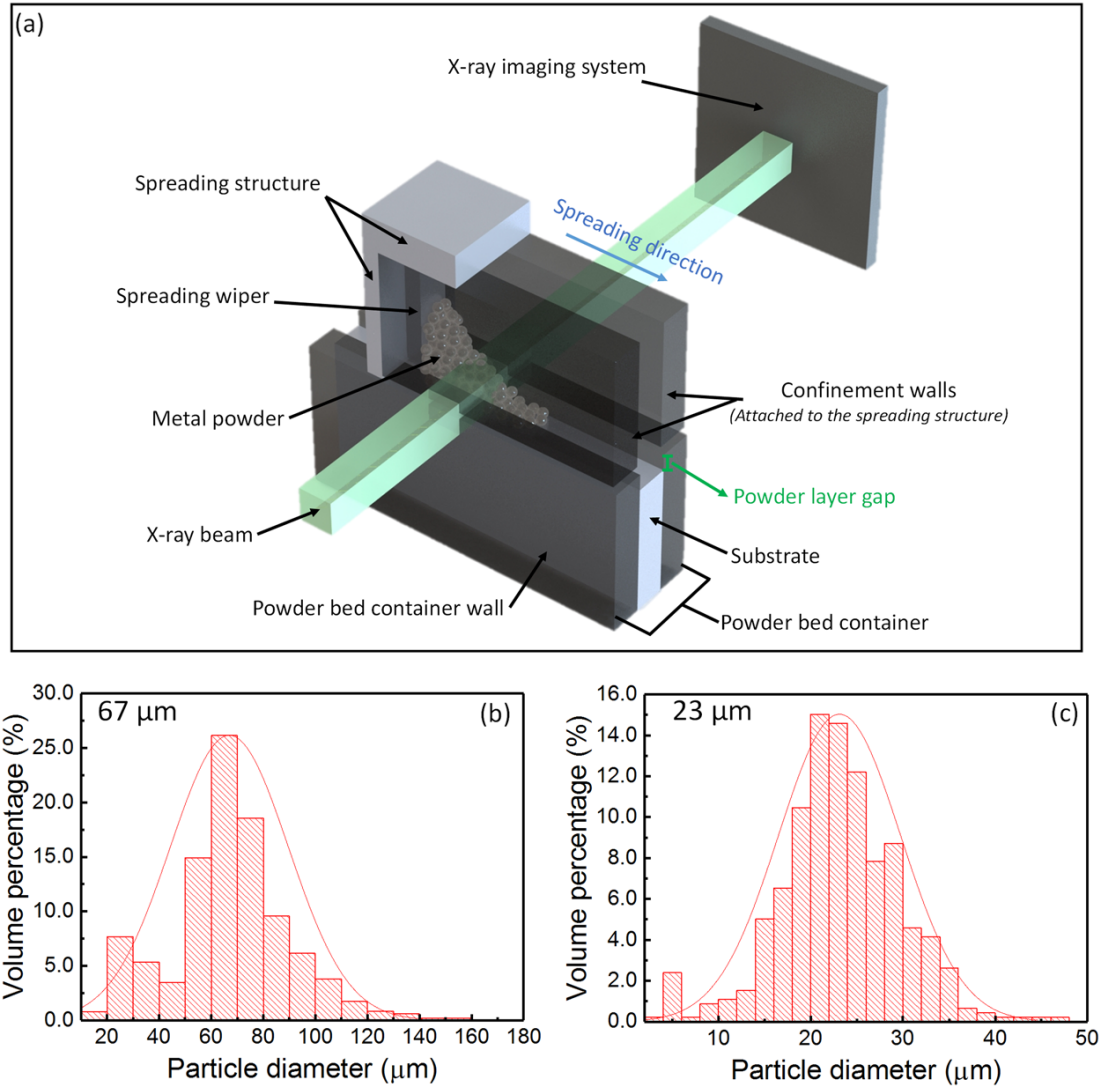
Schematic of the experimental set-up and particle size distributions of 316 L stainless steel powders. (**a**) Schematic of the experimental set-up for *in-situ* powder spreading high-speed x-ray imaging. (**b**,**c**) Powder size distributions for stainless steel powders with average particle diameters of 67 µm (**b**) and 23 µm (**c**). The experiments were carried out at the beamline 32-ID-B of the Advance Photon Source. The experiments were run under an ambient pressure of 1 atm. A rigid aluminum blade with a flat front was used as a wiper. Two rectangular confinement walls were attached to the spreading structure to prevent powders from flowing into path of the x-ray beam. The two powder bed container walls were made of high density graphite to ensure x-ray transparence, low friction and absence of static electric charge accumulation. The powder was spread over an aluminum substrate which was lowered through a z-axis motion stage to create the powder layer gap.

High-speed high-resolution high-energy x-ray imaging was used to overcome the limitations of conventional characterization tools to study the particle-scale powder spreading dynamics. A powder spreading system for *in-situ* x-ray imaging experiments was developed to simulate the spreading process under additive manufacturing configurations, as shown in Fig. 1a.

The *in-situ* experiment system consists of a powder spreading system and x-ray imaging system. The powder spreading system consists of a spreading structure (wiper and confinement walls) and a powder bed container. The spreading wiper is an aluminum blade that is perpendicular to the powder bed substrate. Confinement walls, made of high-density graphite and attached to each side of the aluminum blade, are designed to prevent the powders from flowing out of the spreading system. The powder bed container consists of container walls and a substrate. The container walls are made of high density graphite and the substrate is aluminum. The substrate is controlled by a z-axis motion stage that creates a powder layer gap. The width and length of the powder bed layer are 1 mm and 5 mm, respectively, while the layer thickness is set to be within a range of 80 µm to 160 µm. The low atomic number, low friction coefficient, and good electrical conductivity of the high density graphite ensure that the spreading system has x-ray transparence, low friction, and an absence of accumulated static electric charge.

Powders are spread by an aluminum wiper (blade) at a spreading speed of 11.5 mm/s on an aluminum substrate. As the powder is spread, an x-ray beam passes through the powder bed, and the x-ray signal is recorded by a detection system. The exposure time is 500 ns. A camera is set to record at a speed of 10,000 frames per second and an analysis of the images is done using the ImageJ^29^.

The metal powders used in our experiments are 316 L stainless steel powders of two different average diameters of 67 µm and 23 µm. Particle-size distributions of both powders are shown in Fig. 1b and c. The graphs of particle-size distribution were generated using images of the powders obtained through an optical microscope and then analyzed using ImageJ.

## Results and Discussions

### Dynamic repose angle, dynamic slope surface roughness and slope surface flow speed

The dynamic repose angle, dynamic slope surface roughness and slope surface flow speed are important indicators of powder flow behavior during spreading. However, almost no experimental work has been reported on measuring these parameters during the spreading process under additive manufacturing configurations with high temporal and spatial resolution. With our high-speed high-resolution x-ray imaging method (10,000 frames per second), we characterized, in detail, the dynamic repose angle, the slope surface roughness and the slope surface flow speed of the powder front during the spreading process for 316 L stainless steel powders with two different powder sizes (23 µm and 67 µm).

**Figure 2:**
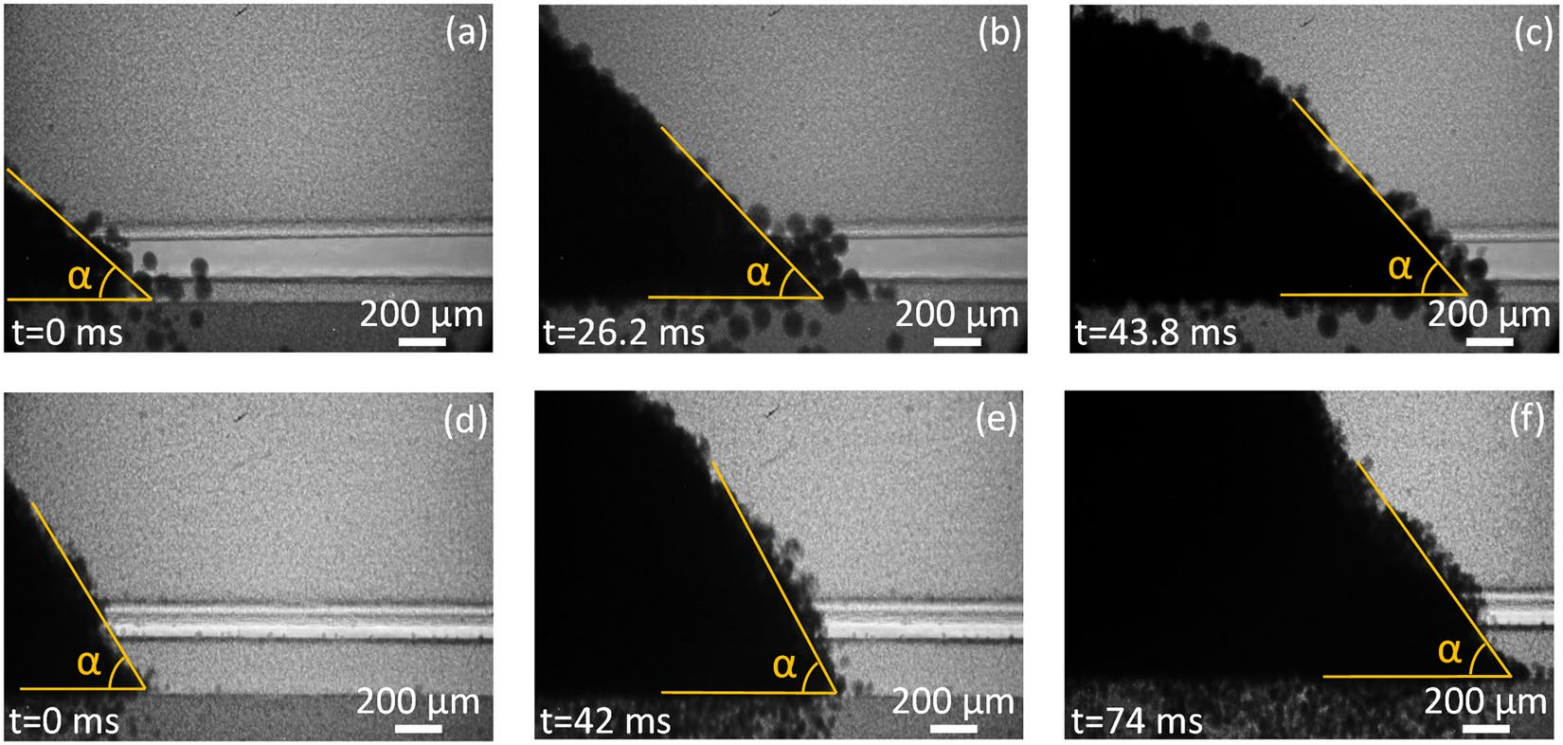
Dynamic x-ray images showing the evolution of dynamic repose angle “α” during the spreading of 316 L stainless steel powders with two different average powder diameters. (**a**–**c**) 316 L stainless steel powder with an average diameter of 67 µm. (**d**–**f**) 316 L stainless steel powder with an average diameter of 23 µm. The two powders were spread with a constant spreading speed of 11.5 mm/s from left to right. A rigid aluminum blade with a flat front (perpendicular to the building platform), was used as the wiper. The powder was spread under an ambient pressure of 1 atm within a stainless steel chamber. The dynamic repose angle “α” of the powder is indicated by the yellow line on each picture.

Figure 2 shows the representative dynamic x-ray images acquired during spreading 316 L powders with average particle sizes of 67 µm and 23 µm. The repose angle and surface roughness at each moment can be clearly observed, which allowed us to conduct a detailed analysis. The repose angle is indicated by yellow lines on each image.

**Figure 3:**
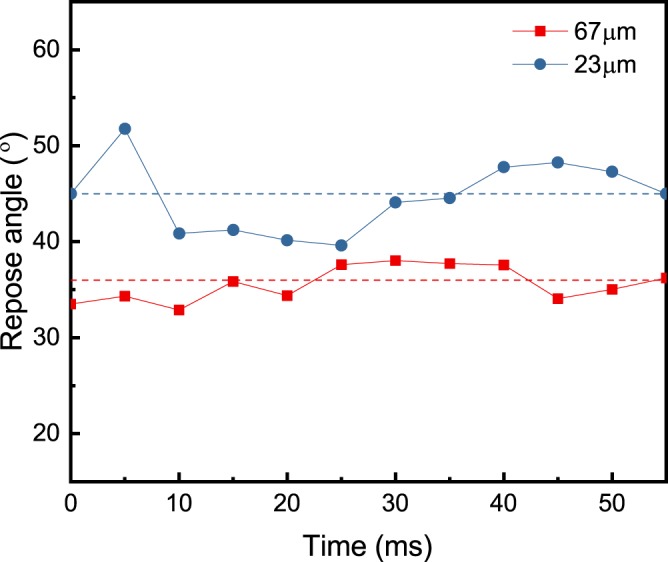
Dynamic repose angle “α” versus time during the spreading of 316 L stainless steel powder with two different average powder diameters. A total of 12 measurements for each powder size was used. There is a time lapse of 5 ms between each measurement. The 316 L stainless steel powder with an average diameter of 67 µm, showed an average dynamic repose angle of 36° with a standard deviation of 2°. The 316 L stainless steel powder with an average diameter of 23 µm showed a higher average dynamic repose angle with a value of 45° and a standard deviation of 4°.

Figure 3 shows the evolution of the repose angle for the two powders over time. The repose angle for 67 µm powder fluctuates in a range of 34° and 37°, with an average value of 36° and standard deviation of 2°. The repose angle for 23 µm of powder fluctuates in a range of 41° and 48°, with an average value of 45° and standard deviation of 4°. The dynamic repose angle data show that the powders with smaller average particle size have a higher dynamic repose angle, larger fluctuation, and a higher standard deviation.

**Figure 4:**
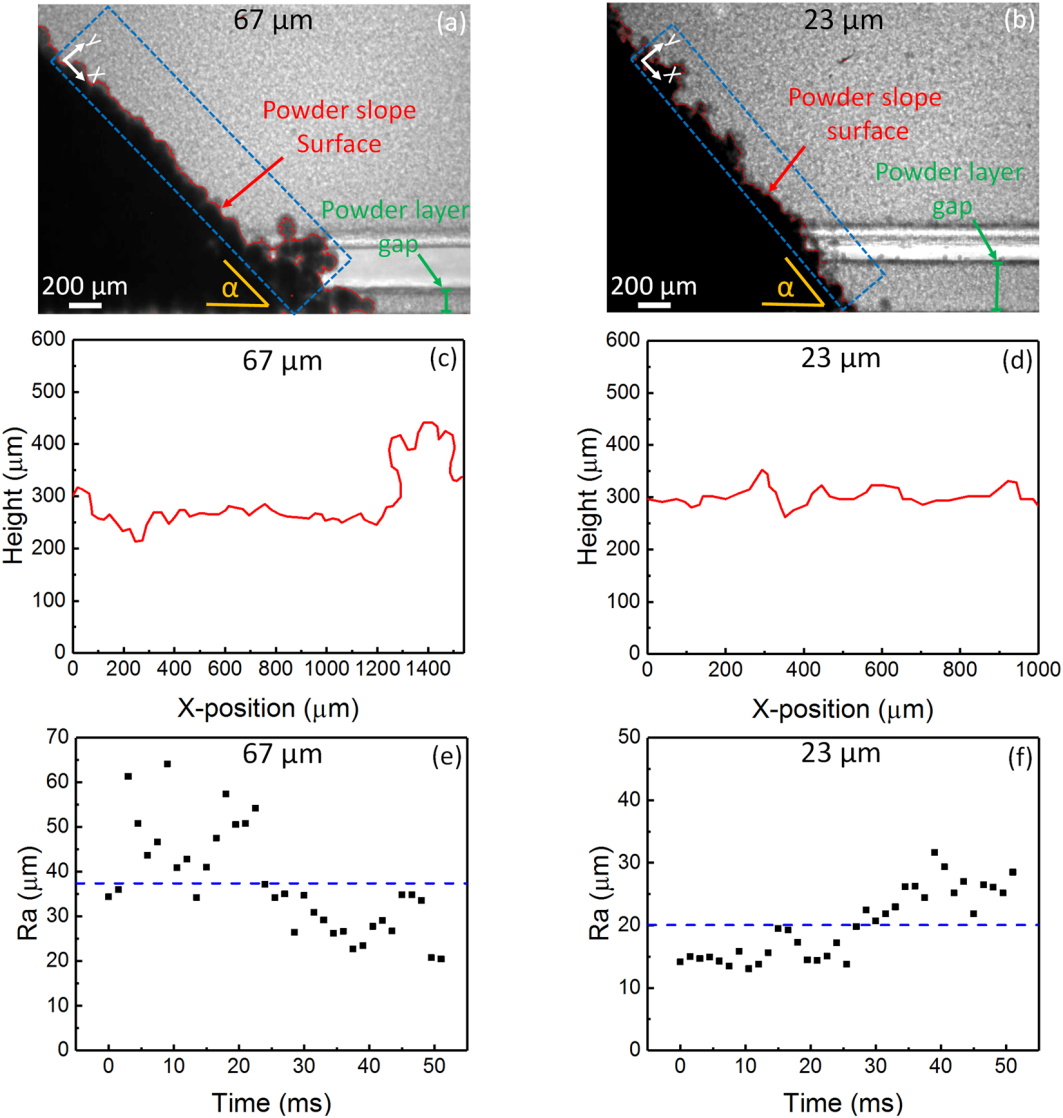
Slope surface roughness. (**a**,**b**) Representative x-ray images showing slope surface fluctuation of powders with an average diameter of 67 µm (**a**) and 23 µm (**b**). (**c**,**d**) Representative slope surface profile of powders with average diameters of 67 µm (**c**) and 23 µm (**d**). Height and X-position are measured with respect to the y-x coordinate shown in (**a**,**b**). (**e**,**f**) Arithmetic average slope surface roughness (Ra) versus time of powders with average diameters of 67 µm (**e**) and 23 µm (**f**). A total of 35 “Ra” measurements were obtained for each powder. A time lapse of 1.5 ms was used between each measurement. The powder with an average diameter of 67 µm showed an average “Ra” of 38 µm with a standard deviation of 11 µm. The powder with an average diameter of 23 µm showed an average “Ra” of 20 µm with a standard deviation of 6 µm.

Figure 4a,b show the representative x-ray images of the slope surface of the powders with average diameters of 67 µm (a) and 23 µm (b). Figure 4c,d depict the representative slope surface profile for each powder. Figure 4e,f show the evolution of the arithmetic average slope surface roughness (Ra) over time for the powders with average diameters of 67 µm (e) and 23 µm (f). A total of 35 measurements of “Ra” were obtained for each powder, using a time lapse of 1.5 ms. The powder with an average diameter of 67 µm shows a larger average “Ra” of 38 µm with a standard deviation of 11 µm; the powder with an average diameter of 23 µm shows a lower average “Ra” of 20 µm with a lower standard deviation of 6 µm. To evaluate the Ra value relative to the average particle diameter, the ratio of Ra/average particle diameter was calculated. The calculated ratio of Ra/average diameter is 0.57 for the powder with an average diameter of 67 µm, which is smaller than the calculated ratio of 0.87 for the powder with an average diameter of 23 µm. The lower ratio of Ra/average particle diameter in larger-sized powder may related to its better flowability.

**Figure 5:**
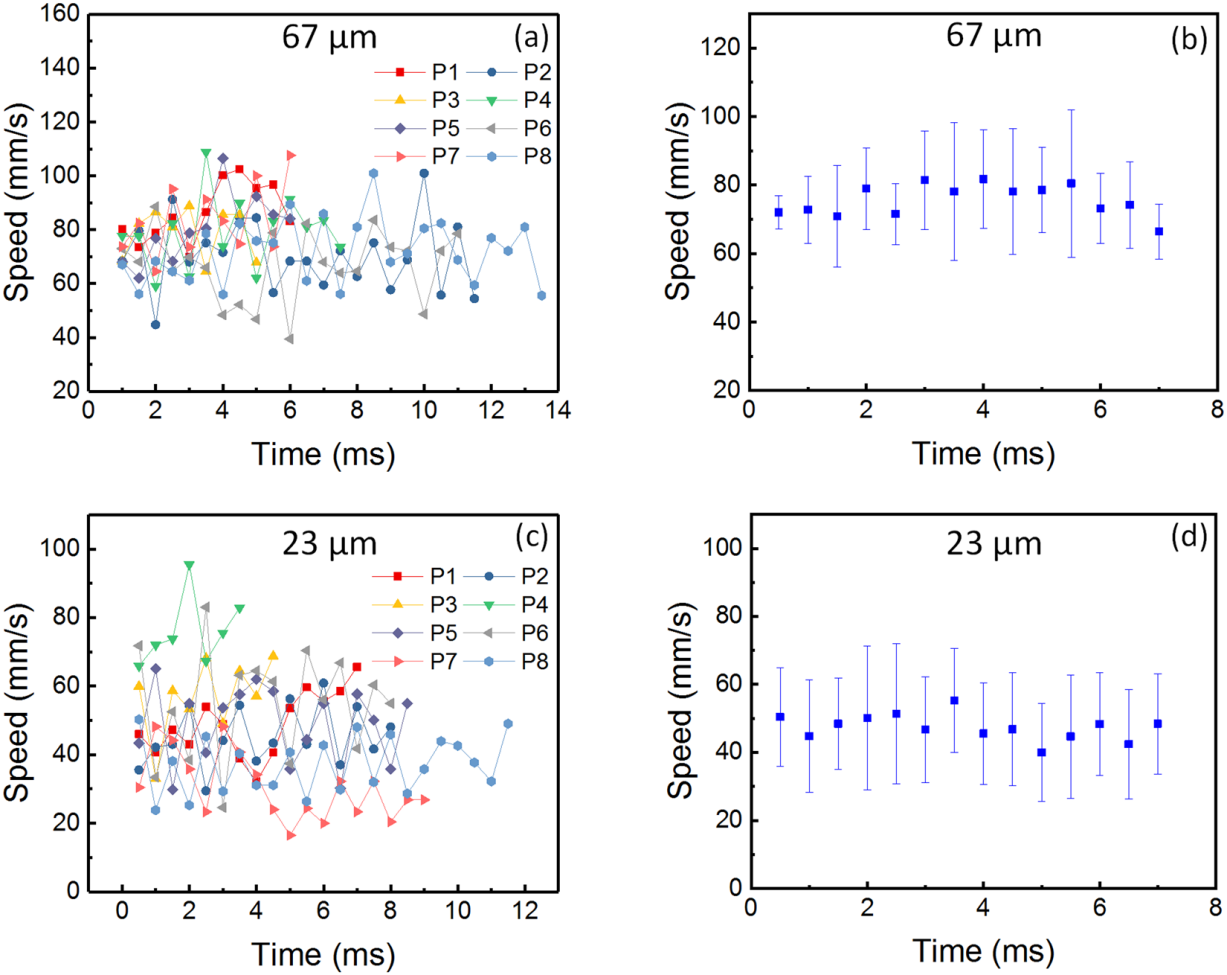
Slope surface flow speed. (**a**,**b**) Slope surface flow speed versus time for each tracked particle (**a**) and average surface flow speed (**b**) for 316 L stainless steel powder with average diameter of 67 µm. (**c**,**d**) Slope surface flow speed versus time for each tracked particle (**c**) and average surface flow speed (**d**) for 316 L stainless steel powder with an average diameter of 23 µm. Eight particles on the surface of the powder slope were tracked for each powder size. The 316 L stainless steel powder with an average diameter of 67 µm presented an average slope surface flow speed of 77 mm/s with a standard deviation of 7 mm/s. The 316 L stainless steel powder with an average diameter of 23 µm presented an average slope surface flow speed of 50 mm/s with a standard deviation of 14 mm/s.

Figure 5 shows the evolution of the slope surface speed over time for the two powders. A total of eight particles, flowing on the slope surface, were tracked. A time step of 0.5 ms was used between each measurement. Figure 5a,c show the speed over time of the tracked particles for powders with average sizes of 67 µm and 23 µm. Figure 5b,d show the results of the calculated average slope speed and variations in time for powders with average sizes of 67 µm and 23 µm. The 67 µm of powder showed an average slope surface speed of 77 mm/s that fluctuated within a range of 70 mm/s and 84 mm/s, while the 23 µm of powder showed an average surface flow speed of 50 mm/s that fluctuated within a range of 36 mm/s and 64 mm/s.

The reduced slope surface flow speed and higher flowing variations shown by powder with a smaller average size, was consistent with the instabilities found in the dynamic repose angle. This confirmed the reduction in flowability and increased flow irregularities as the average size of the powder was reduced. The large variation in the slope surface flow speed in the 23 µm powder might contribute to the larger ratio of Ra/average particle diameter observed for this powder.

### Dynamics of powder clusters

A common instability during powder spreading in powder-bed-based additive manufacturing process is the formation of powder agglomerations, also known as powder clusters. The clusters flow in a different way through the powder slope surface, when compared to individual particles. Therefore, the study of their flowing behavior is important for characterizing the state of the powder that has flowed into the layer gap. Unfortunately, almost no experimental data has been reported about the flowing behavior and evolution of powder clusters. It is also difficult for DEM simulations to accurately simulate the formation of powder clusters during the spreading process. By using our high-speed high resolution x-ray imaging method, we conducted an *in-situ* analysis of the behavior and evolution of two types of clusters flowing through the slope surface during the spreading of 316 L stainless steel powder with an average size of 67 µm.

**Figure 6:**
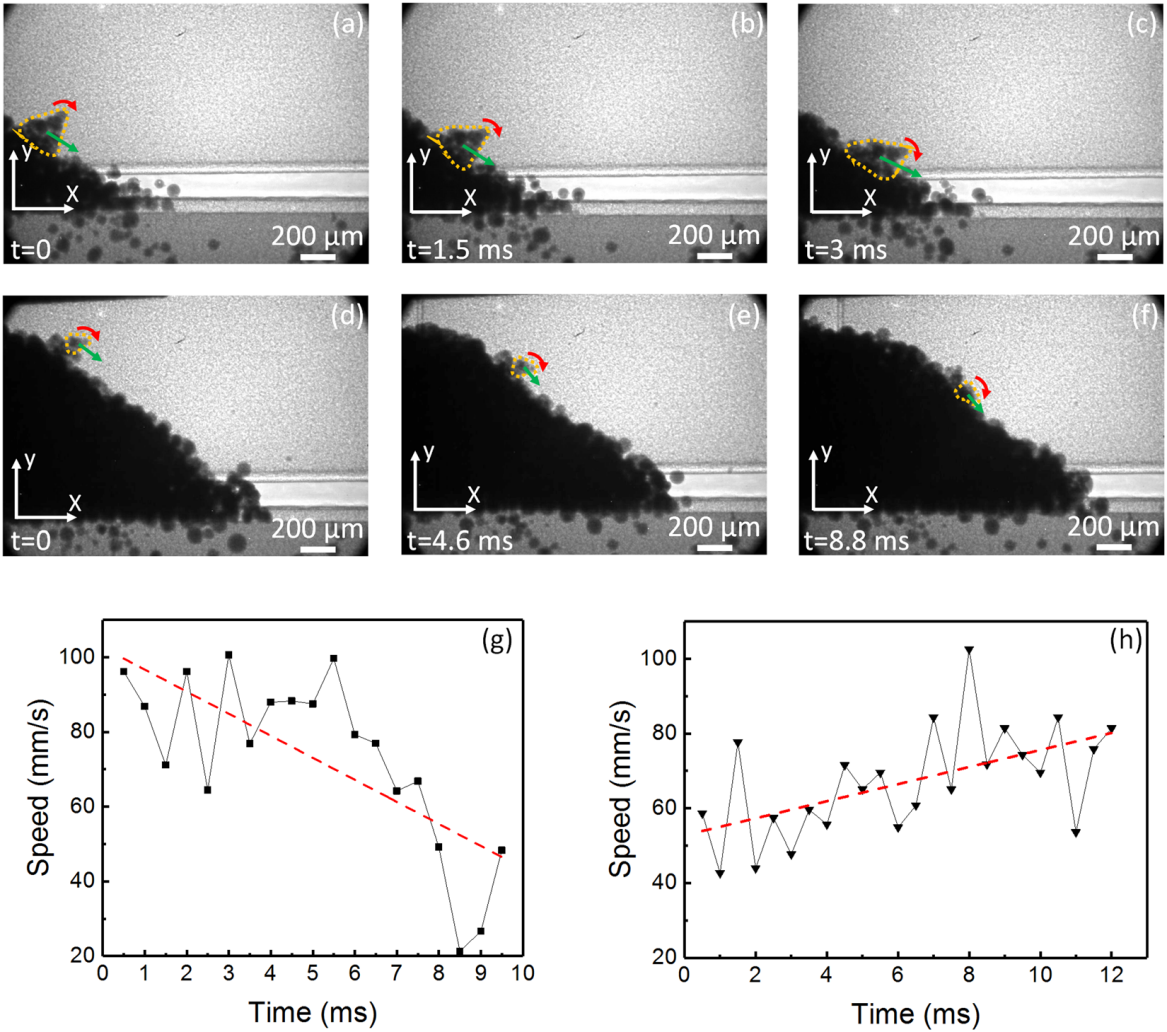
Dynamic evolution of powder clusters tracked on the surface of the slope during the spreading of 316 L stainless steel powder with an average particle diameter of 67 µm. (**a**–**c**) Dynamic x-ray images showing the position and evolution of the soft powder cluster “A”. (**d**–**f**) Dynamic x-ray images showing the position and evolution of the hard powder cluster “B”. The yellow dotted line highlights the cluster’s boundary. The green line indicates the current translational trajectory. The red line indicates the current rotational trajectory. The position was tracked with reference to the origin of the coordinate system in each picture. (**g**) The speed versus time for cluster “A”. (**h**) The speed versus time for cluster “B”. The red dashed line is the linear fitting of each graph. The cluster “A” presented an average flow speed of 73 mm/s with a standard deviation of 23 mm/s. The cluster “B” presented an average flow speed of 67 mm/s with a standard deviation of 15 mm/s.

Figure 6 shows representative images of the evolution of the tracked clusters as they flowed through the slope surface. Figure 6(a–c) shows the evolution of the first cluster, designated as cluster “A”. Figure 6(d–f) shows the evolution of the second cluster, designated as cluster “B”. The high quality of these images allow us to precisely track the position and morphologic changes of each cluster. The cluster structure and its translational and rotational directions are indicated by the yellow, green, and red lines, respectively. It can be noticed that cluster “A” changes its structural configuration as it flows through the slope surface, in contrast with cluster “B” which keeps a constant morphologic configuration. Therefore, cluster “A” was identified as a “soft cluster”, and cluster “B” as a “hard cluster”. The position of each cluster, as a function of time, was tracked and their speed (over time) was computed and plotted.

Figure 6g,h shows the speed over time for the tracked powder clusters “A” (soft cluster) and “B” (hard cluster) respectively. A time lapse of 0.5 ms was used between each data point. The red dashed line indicates the linear fitting on each plot. Cluster “A” presented an average flow speed of 73 mm/s, with a standard deviation of 23 mm/s, that fluctuated within a range of 50 mm/s and 96 mm/s, while cluster “B” presents an average flow speed of 67 mm/s, with a standard deviation of 15 mm/s, that fluctuated within a range of 52 mm/s and 82 mm/s. Compared to the data obtained for the slope surface flow speed of this powder, cluster “A” shows a 5 mm/s slower average flow speed and had an increased standard deviation by 16 mm/s. Cluster “B” shows a 11 mm/s slower average flow speed and had an increased standard deviation of 8 mm/s. This data indicate the instability that powder clusters added to the powder flow on the slope surface of the powder front.

**Figure 7:**
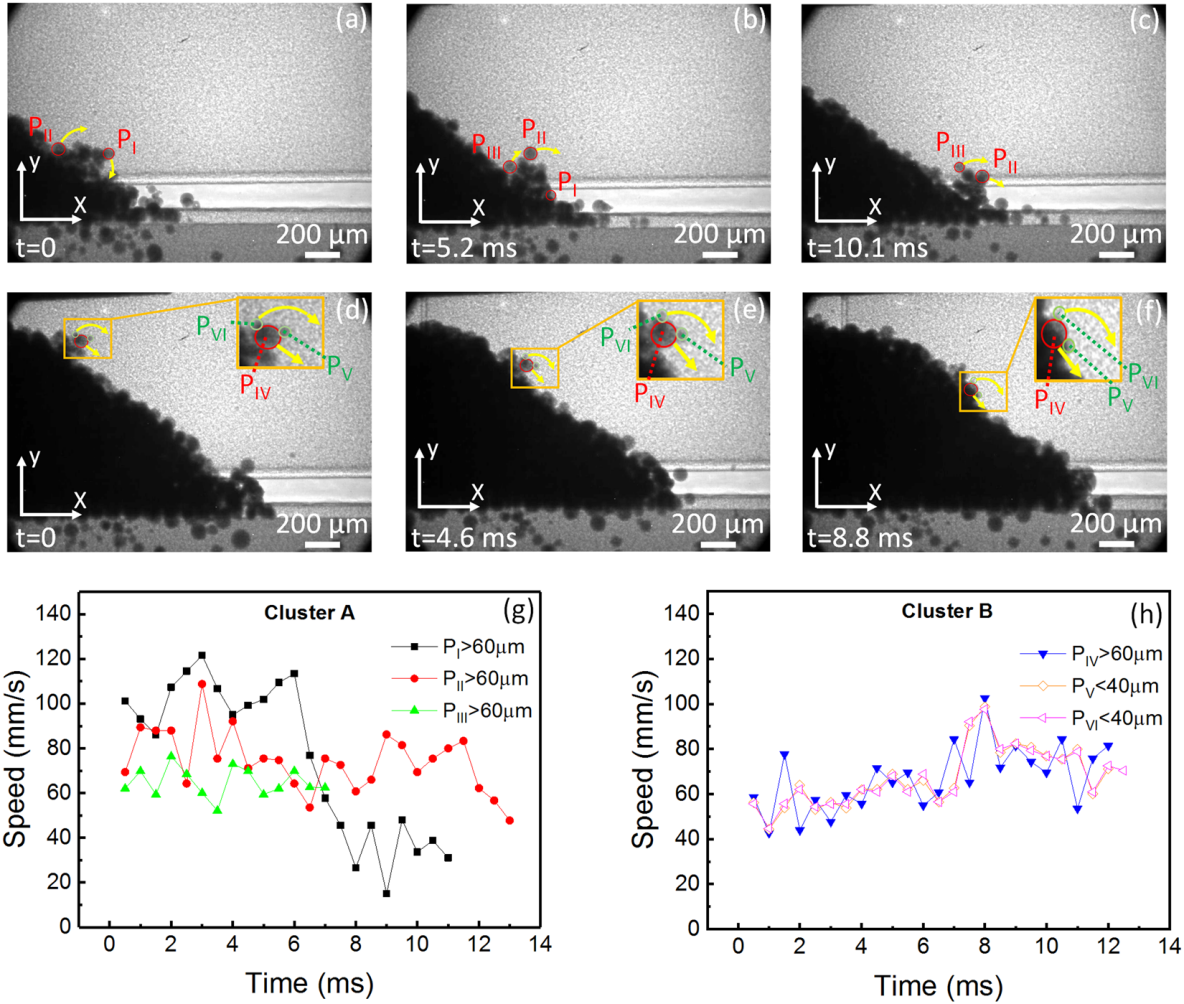
Tracking of individual particles of different sizes within the powder clusters during the spreading of 316 L stainless steel powder with an average diameter of 67 µm. (**a**–**c**) Dynamic x-ray images showing the motion of particles P_I_, P_II_ and P_III_ within the soft powder cluster “A”. (**d**–**f**) Dynamic x-ray images showing the motion of particles P_IV_, P_V_ and P_VI_ within the hard powder cluster “B”. Yellow arrows show the translational and rotational trajectory of the powders. The position versus time was tracked with reference to the origin of the coordinate system in the pictures. (**g**) Speed over time for particles within “cluster A”. (**h**) Speed over time for particles within “cluster B”. (P_I_–P_III_, P_IV_) are particles with a diameter more than 60 µm. (P_V_–P_VI_) are particles with a diameter smaller than 40 µm.

For a deeper understanding of the flow behavior of the powders within the powder clusters, a particle scale analysis was conducted. Figure 7 shows representative images of the particle scale tracking for each of the powder clusters. Figure 7(a–c) show the evolution of the particles found within cluster “A”. Figure 7(d–f) show the evolution of the particles found within cluster “B”. Particles larger than 60 µm and smaller than 40 µm are highlighted in each image by red and yellow circles, respectively. The rotational direction of each particle is represented by the yellow arrows on each image. The position, as a function of time, of each particle was tracked and the speed over time was computed and plotted.

Figure 7g,h show the plotted speed over time of the tracked particles within each cluster. Figure 7g shows the speed over time of the particles belonging to the soft cluster (cluster “A”), while Fig. 7h shows the speed over time for the particles within the hard cluster (cluster “B”). A total of six particles were tracked, (P_I_, P_II_, P_III_) within cluster “A”, and (P_IV_, P_V_, P_VI_) within cluster “B”. On a particle scale, Fig. 7g show substantially different relative speed variations for particles P_I_, P_II_, and P_III_ belonging to cluster “A”. However, particles belonging to cluster “B” (P_IV_, P_V,_ and P_VI_) showed relatively similar speed variations in Fig. 7h.

The formation of a soft cluster may result from van der Waals cohesion force and/or particle packing-induced jamming. A hard cluster may be a powder with satellites (formed in the atomization process) or may form due to a much stronger van der Waals cohesion force, as compared to gravity force, because of the large difference in diameters. The irregular surfaces of these kinds of morphologies restrict the free flow of powder during the spreading process. With this restricted flow, the powders tend to jam and create an irregular powder slope surface (shown in Fig. 7). With this unstable flow, it might cause formation of cavities and voids in the powder bed.

### Interaction of powders with boundaries and calculation of coefficients of friction

Metal powders interact with the substrate and container walls during the powder spreading process. However, an analysis of these interactions using an experimental approach is challenging. As a consequence, almost no experimental data has been reported on the analysis of the physical interactions between powder and the boundary surfaces at particle scale. Our high-speed x-ray imaging method allowed us to analyze the dynamics of two individual particles as they flowed away from the powder front over two different surfaces. The first particle was found to flow over the aluminum substrate, while the second particle flowed on the surface of the graphite container walls.

**Figure 8:**
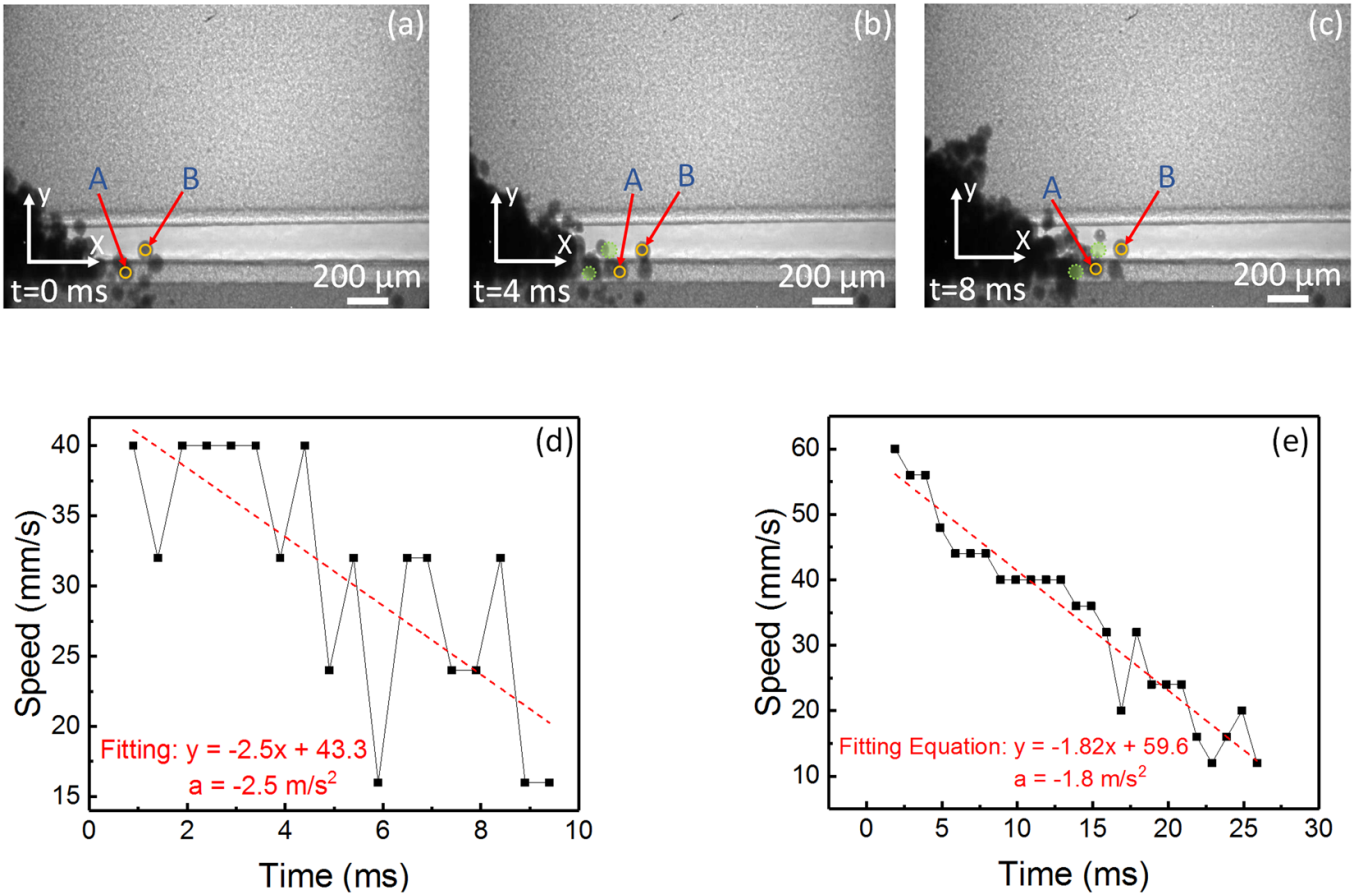
Tracking of individual particles motion over substrates during the spreading of 316 L stainless steel powder with average diameter size of 67 µm. (**a**–**c**) Dynamic x-ray images showing the motion of particles (particle A and particle B) moving on the substrates during the spreading of 316 L stainless steel powder with an average powder diameter of 67 um. Particle “A” is moving over a flat ground aluminum surface that serves as substrate for the process. Particle “B” is moving over a flat ground graphite surface that served as container wall for the powder bed. The green dotted spheres represent the previous position. The position versus time was tracked with reference to the origin of the coordinate system in the pictures. (**d**) Translational speed versus time of particle “A”. (**e**) Translational speed versus time of particle “B”. The red dotted lines are the linear fitting of each graph. The equations shown are the fitting equations of fitting lines. The first differential of each fitting equation provides the acceleration of the powder. The resulting translational acceleration of particles A and B are −2.5 m/s^2^ and −1.8 m/s^2^, respectively.

Figure 8(a–c) illustrates representative dynamic x-ray images showing the motion of the two particles. The previous and current position of each particle is indicated by the green and yellow circles, respectively. The particle flowing over the aluminum substrate was designated as particle “A”, while the other one, flowing over the graphite container wall surface, was designated as particle “B”. The position of each particle, as a function of time, was tracked until their movements had ceased due to friction. The speed over time was then computed, along with the average acceleration.

Figure 8d,e show the speed versus time plots of the tracked particles “A” and “B”. The red dashed line indicates the linear fitting on each plot. The corresponding linear fitting equations are also shown. The first derivative of each equation provides the value of the current acceleration experienced by each particle. The computed acceleration for particle “A”, as it moved over the aluminum surface, was −2.5 m/s^2^, while the computed acceleration for particle “B”, as it moved over the surface of the high-density graphite wall, was −1.8 m/s^2^.

Using Newton’s second law of motion, the corresponding coefficients of friction could be obtained. For the current translational movement, the force suffered by the particles is the friction force “F_c_”. The kinetic friction coefficient could be obtained as:

1 $${\mu }_{c}=\frac{ma}{N}$$

where “${\mu }_{c}$” is the kinetic coefficient of friction, “m” is the mass of the particle ($2.1\,\times \,{10}^{-9}Kg$ for both particles), “a” is the acceleration of the particles (−2.5 m/s^2^ and −1.8 m/s^2^ for particle A and B, respectively) and “N” is the normal force applied by the surface to the particle ($2.1\,\times \,{10}^{-8}N$ for both particles). The calculated kinetic friction coefficients are 0.25 for particle “A” moving over aluminum substrate, and 0.18 for particle “B” moving over high density graphite ground surface.

## Conclusions

Particle-scale powder spreading dynamics in powder-bed-based additive manufacturing process was revealed by high-speed high-energy x-ray imaging. The major conclusions are summarized below.

1. A high-speed high-resolution high-energy x-ray imaging approach was developed that enables the study of the particle-scale powder spreading dynamics in the powder-bed-based additive manufacturing process. Evolution of the slope surface speed, slope surface roughness, and dynamics of powder clusters at the powder front are revealed and quantified.
2. The average powder size is an important parameter that affects powder flow dynamics during the spreading process. The powder with a larger average diameter of 67 µm showed a higher average dynamic repose angle of 36°, and a higher average slope surface flow speed of 77 mm/s, as compared with the powder with a smaller average diameter of 23 µm, that showed an average dynamic repose angle of 45°, and an average slope surface flow speed of 50 mm/s.
3. Powder clusters affect powder spreading behavior. The powder clusters could not easily flow through the slope surface.
4. Interactions of powders with boundaries were characterized and the coefficients of friction were calculated. The calculated kinetic coefficients of friction were 0.25 for particle moving over aluminum substrate, and 0.18 for particle moving over a high density graphite surface, which shows the potential for using the developed *in-situ* x-ray imaging approach to get critical boundary condition parameters for DEM modeling.

The particle-scale powder spreading dynamics revealed are important for understanding powder spreading behavior in the powder-bed-based additive manufacturing process. This is critical for the development and validation of more accurate models for predicting powder spreading behavior.

## Methods

### Materials

316 L stainless steel powders of two different average diameters of 67 µm and 23 µm were used for this study. The 316 L stainless steel powders were produced using gas atomization method.

### Substrate, spreading wiper, container walls and confinement walls

The substrate, spreading wiper, container walls and confinement walls were machined from a 6061 aluminum block and high density graphite block using a milling machine (Sherline 5810 NexGen). The surfaces were then polished by hand using an 800 (P-2400) silicon carbide metallurgical abrasive paper. Before the experiments, the structures were cleaned using wipers and alcohol.

### X-ray imaging system

The x-ray imaging system consists of an x-ray beam and detection system. The x-ray beam used is an undulator-generated pink beam with the harmonic energy set to 25.7 keV and an energy bandwidth of 5~7% (Beamline 32-ID-B at the Advanced Photon Source). The x-ray detection system mainly consists of a scintillator (LuAG:Ce, 100 µm thickness) and a high-speed camera (Photron FastCam SA-Z).

### Powder size distribution

A total of 8 samples were extracted and analyzed for each 316 L stainless steel powder. Each sample was carefully spread over a glass substrate, then images were obtained using an optical microscope. “Analyze Particles” tool in ImageJ was used for the computation of the particle diameter. A total of 2700 particles for each powder where analyzed. The powder size distribution graphs were then generated based on volumetric percentage.

## Data Availability

The datasets generated during and/or analyzed during the current study are available from the corresponding author on reasonable request.
